# The lymphatic vascular system: much more than just a sewer

**DOI:** 10.1186/s13578-022-00898-0

**Published:** 2022-09-15

**Authors:** Jörg Wilting, Jürgen Becker

**Affiliations:** Department of Anatomy and Cell Biology, University Medical School Göttingen, Göttingen, Germany

**Keywords:** Initial lymphatics, Lymphatic collector, Lymphangiogenesis, Circulating endothelial precursor cells, Pacemaker cell, Smooth muscle cell origin, Sphingosine-1-phosphate, Melanocortin-2 receptor accessory protein-2

## Abstract

Almost 400 years after the (re)discovery of the lymphatic vascular system (LVS) by Gaspare Aselli (Asellius G. De lactibus, sive lacteis venis, quarto vasorum mesaraicorum genere, novo invento Gasparis Asellii Cremo. Dissertatio. (MDCXXIIX), Milan; 1628.), structure, function, development and evolution of this so-called ‘second’ vascular system are still enigmatic. Interest in the LVS was low because it was (and is) hardly visible, and its diseases are not as life-threatening as those of the blood vascular system. It is not uncommon for patients with lymphedema to be told that yes, they can live with it. Usually, the functions of the LVS are discussed in terms of fluid homeostasis, uptake of chylomicrons from the gut, and immune cell circulation. However, the broad molecular equipment of lymphatic endothelial cells suggests that they possess many more functions, which are also reflected in the pathophysiology of the system. With some specific exceptions, lymphatics develop in all organs. Although basic structure and function are the same regardless their position in the body wall or the internal organs, there are important site-specific characteristics. We discuss common structure and function of lymphatics; and point to important functions for hyaluronan turn-over, salt balance, coagulation, extracellular matrix production, adipose tissue development and potential appetite regulation, and the influence of hypoxia on the regulation of these functions. Differences with respect to the embryonic origin and molecular equipment between somatic and splanchnic lymphatics are discussed with a side-view on the phylogeny of the LVS. The functions of the lymphatic vasculature are much broader than generally thought, and lymphatic research will have many interesting and surprising aspects to offer in the future.

## Introduction

In contrast to the cardiovascular system, studies on the lymphatic system are significantly fewer. The perceived importance of the cardiovascular system is most likely due to the fact that diseases of this system are associated with a high mortality rate. In contrast, (primary) diseases of the lymphatic vascular system are *‘only’* life-long disabling and disfiguring, but rarely lethal. Among the potentially lethal lymphatic diseases, the protein-losing enteropathy (PLE) must be mentioned. PLE can be primary (cystic hypoplasia of intestinal lymphatics) or secondary (diseases that block the function of the intestinal lymphatic system) [2, 3], and causes multiple life-threatening sequelae such as lymphopenia with immunodeficiency, and septicemia. In some vertebrate species, failure of lymphatic system development and function can be fatal. In embryonic birds for example, sacral lymph hearts drain fluid from the lower parts of the body and the extraembryonic membranes into coccygeal veins. Blockage of lymph hearts or their aplasia (in Araucana rumples chicken) cause embryonic edema with fatal outcome [4]. Similarly, in humans, fatal embryonic edema is found in non-immune hydrops fetalis, which, among others, can be caused by congenital lymphatic dysplasia [5]. The subcutaneous tissue is characterized by wide intercellular spaces and multiple cysts lined by D2-40/Podoplanin-positive lymphatic endothelial cells (LECs). Mutations in the *FOXC2* forkhead-family transcription factor have been found [5], which is highly expressed in the jugular region where the jugular lymph sacs develop, as well as a valve that prevents backflow of blood into lymphatics [6]. Later in development, FOXC2 controls the formation of intraluminal valves in lymph collectors [7].

**Fig. 1:**
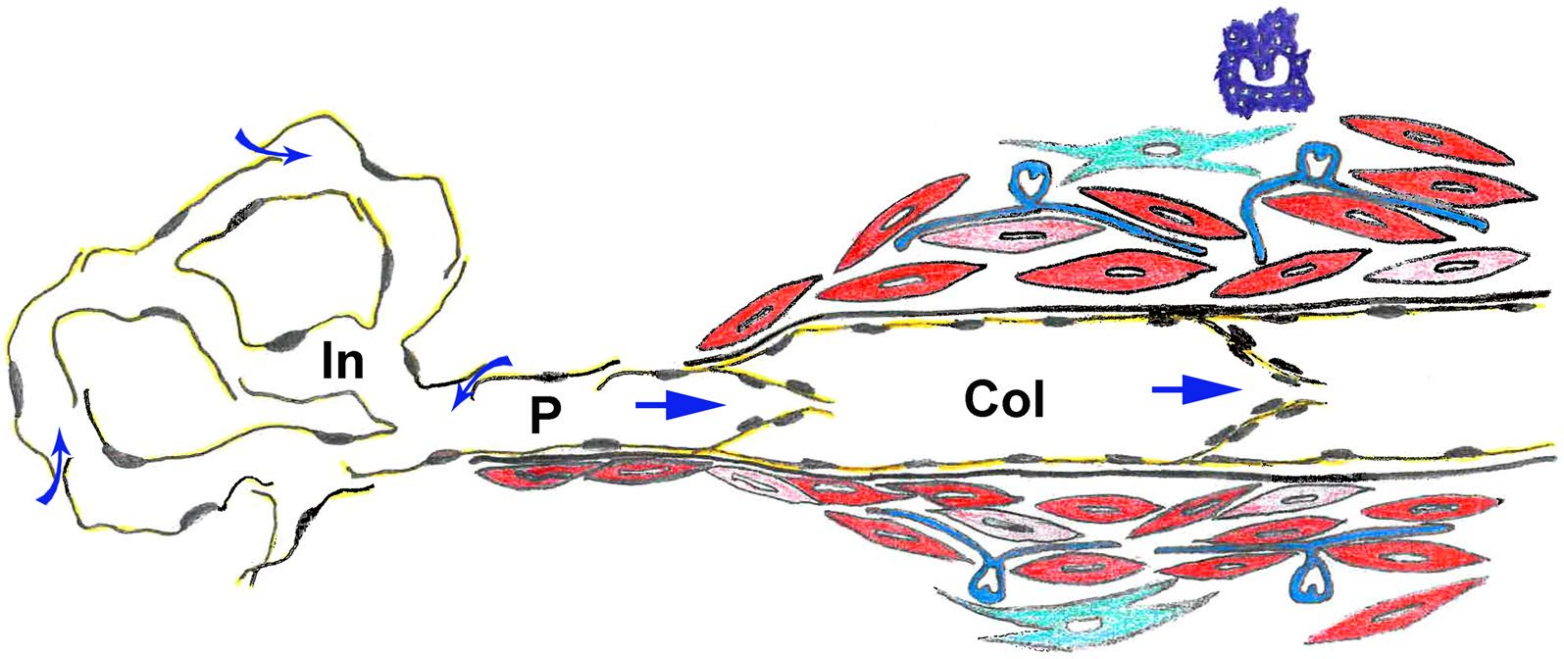
Scheme of the conventional cellular composition of the lymphatic vascular system. Initial lymphatics (In) are composed of LECs (yellow) with microvalves that allow influx of interstitial fluid (curved arrows). Precollectors (P) have a mixed phenotype of both initial lymphatics and collectors (Col). The latter possess a continuous basement membrane underneath the LECs. Their wall is made up of dark and light smooth muscle cells (red), fibrocytes (green), interstitial Cajal-like pacemaker cells (blue), and mast cells (violet). The vegetative innervation of collectors and the *vasa vasorum* of the particularly muscular collectors are not shown. Straight blue arrows indicate the direction of lymph flow regulated by intraluminal valves

Malformations of the lymphatic vascular system can affect any part of the system: initial lymphatics (capillaries), precollectors, collectors (schematically shown in Fig. 1), lymph nodes, trunks and the lympho-venous connections. This causes lymphatic insufficiency and primary lymphedema (LE). Systematic studies on primary LE are very rare. The incidence of primary LE at birth is (estimated) about 1:6000 [8], and the prevalence is about 1:87,000 in those under 20 [9]. Thereby, the female sex is affected 5.5 times more often than the male sex, and the inguinal region is significantly (18.5 times) more often affected than the axillary region [10].

Tumors that originate from LECs are very rare. Surprisingly, the Hobnail ‘hemangioma’ is a benign tumor of the mid-dermis which appears to be a lymphangioma. The tumor cells are positive for the LEC marker D2-40/Podoplanin and negative for the blood endothelial cell (BEC) marker CD34, which characterizes them as LECs [11]. Besides Kaposi sarcoma, various types of cutaneous angiosarcomas have been described as endothelium-derived tumors. Lymphedema-associated angiosarcoma (Stewart-Treves Syndrome) is a very rare but highly malignant tumor that may be derived from LECs [12–14].

It has become increasingly clear lately that the function of the lymphatic vascular system goes beyond fluid homeostasis. In this review, we give an outline of the structure and function of the lymphatics. We will attempt to illustrate the diversity of functions of the lymphatic vasculature, and refer to the influence of hypoxia, a characteristic of lymphedema. Molecular heterogeneity of LECs in the different sections of the system, and the diversity of their development and function in the body wall (somatic) and internal organs (splanchnic) are discussed in terms of malfunctioning.

## Structure of the lymphatic vascular system

There are very few organs which lack both blood vessels and lymphatics, such as the cornea, which expresses high amounts of inhibitors of angiogenesis and lymphangiogenesis [15]. Lymphatics are absent in organs and tissues that are protected by an immunological barrier, such as the blood–brain and the blood-nerve-barrier. Otherwise, they are present in practically all organs, although their total length clearly does not reach the 50,000 to 100,000 km of the blood vascular system [16]. Lymphatics usually follow the course of arteries and finally drain into the left and right ‘venous angle’ in the neck, originally described by Rudbeck [17]. However, more than these two lympho-venous anastomoses seem to exist (for discussion see: Ref. [18]. At the base of the heart and in the epicardium, lympho-venous anastomoses have been found [18, 19]. In monkeys, lymphatics were described to connect to the renal veins [20]. Fine endothelial cell protrusion without any lumen connecting blood capillaries and initial lymphatics have been observed in rat mesenteric tissue [21]. However, functional data are missing.

### Initial lymphatics

**Fig. 2:**
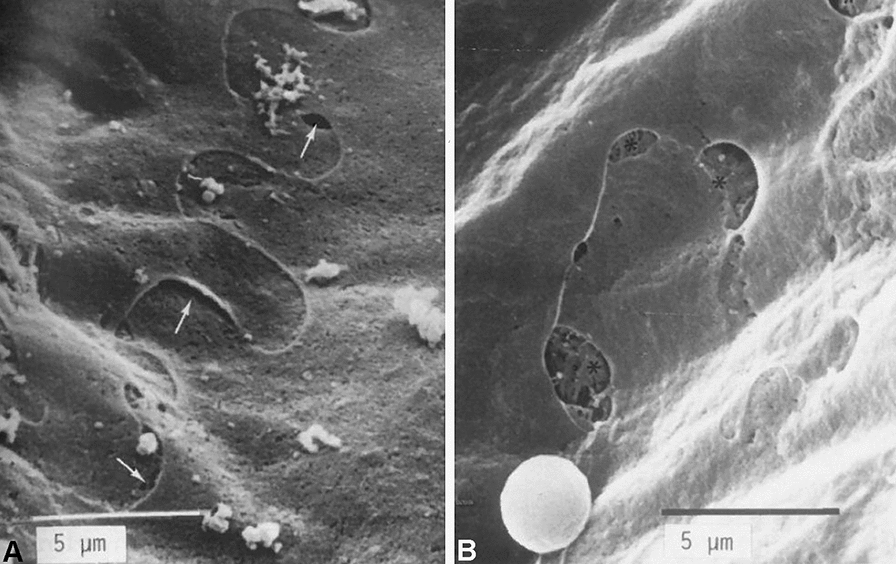
Scanning electron microscopy of the luminal surface of initial lymphatics in rats. **A** Note the oak-leaf shaped overlapping cell protrusions of the LECs. **B** Due to shrinkage, the extracellular matrix beneath the LECs has become visible. Pictures taken from: [24]; with permission from the Gesellschaft Deutschsprachiger Lymphologen. Bar = 5 µm

The endothelial lining of the initial lymphatics (often called capillaries) (Fig. 1) was already described by Recklinghausen [22], a student of Rudolph Virchow, using the emerging microscopic techniques and silver impregnation methods. In his paper, he confirms the nodular thickenings (valve segments) of lymphatic vessels (collectors) observed earlier, the spindle-shaped form of the epithelial cells (now called endothelial cells) of the collectors, and the absence of smooth muscle cells in the fine branches. He then described that an epithelium (today endothelium) can still be seen in the finest branches of the lymphatic vessels; e.g. in the chylous vessels of the intestinal villi and the initial lymphatic vessels of the diaphragm. Histological images of LECs using silver impregnation methods have been carried out repeatedly, and convincingly show the oak-leaf shape of the LECs of initial lymphatics [23]. The typical shape of these cells was also demonstrated with scanning electron microscopy (Fig. 2; [24]). There has been a fierce debate about the function of the broad overlapping protrusions of the LECs. Of note, due to shrinkage of histological specimens, the overlapping parts of the protrusion are often retracted, holes appear, and the cells look more like “interlocked”. Castenholz [24] suggested that overlapping protrusions are ‘Einwegklappen’ (one-way valves) opened by active contractions of the LECs thereby producing holes between the LECs (as depicted in Fig. 2B) [24]. It was also suggested that the peri-endothelial extracellular matrix (ECM), especially the anchoring filaments (first describe by Leak and Burke) [25], exert an outward pull on the flaps allowing fluid flow into the lymphatics. However, anchoring filaments are mainly made up of fibrillin-1 [26], an elastic molecule that should be able to compensate for slight changes in distance between LECs and the ECM. It is now well accepted that the micro-valves are regulated by delicate pressure gradients. They possess a mobile part that opens towards the lumen of the vessel flanked by two ‘button-like’ hinges, which are stabilized by VE-cadherin and various tight-junction molecules [27, 28]. It is the micro-valves in the initial lymphatics that prevent backflow of lymph into the interstitium [27].

### Lymphatic collectors

**Fig. 3:**
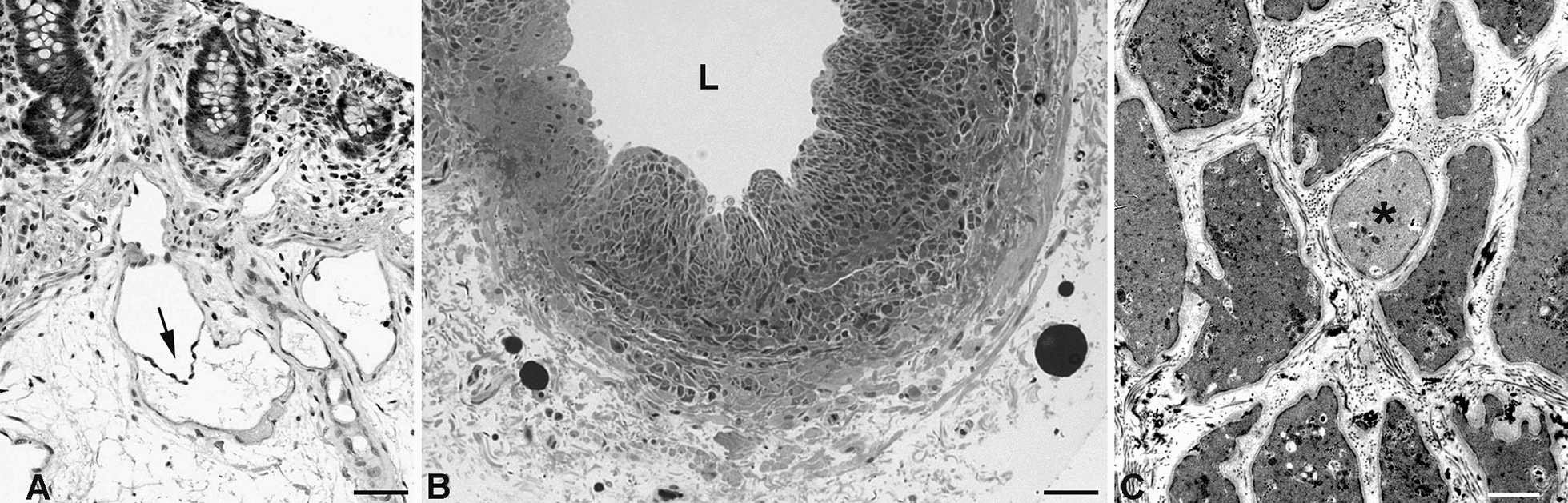
**A** Human Colon. Transition from the initial lymphatic in the basal mucosa to the precollector in the submucosa. Note the intraluminal valve in the precollector (arrow). (From: The human protein atlas [213]. **B** Human lymphatic collector from the hypodermis of the thigh. Note the strong muscular wall. L = lumen of the collector. **C** Transmission electron microscopy shows that the smooth muscle cells usually appear as electron-dense dark cells, while some have a light cytoplasm (asterisk). Bar = 45 µm in A, = 150 µm in B, and 4 µm in C

Initial lymphatics are followed by lymphatic collectors (Fig. 1). In lymphatic collectors, LECs are (almost) tightly sealed by continuous formations of tight-junctions, described as ‘zipper-like’, making collectors highly impermeable [28]. In between there is a vascular segment that has characteristics of both initial lymphatics and collectors, and is called precollector (Fig. 1). Precollectors may already possess intraluminal, bicuspid valves (Fig. 3A), but this does not completely prevent backflow of lymph upon contraction of the collectors.

Lymphatic collectors form a network of interconnected hierarchical channels. Thereby, highways exist as well as alternative routes with correspondingly adapted thickness of the muscular wall. Usually, with life imaging techniques (e.g. lymph-scintigraphy or indocyanine green lymphangiography) only the highways will show up [29], unless they are blocked. In the human, it is likely that the strongest lymphatic collectors are present in the legs. The contractile strength of the collectors was measured with blood pressure cuffs in the arm. There, the average occlusion pressure was found to be 56 mmHg [30] or 86 mmHg [31]. The view of the lymphatics as a low-pressure system is wrong. The muscular wall of collectors can be very strong (Fig. 3B) and is then supplied by *vasa vasorum* [32]. Contractility of lymphatics was described by William Hewson already in 1772 [33].

**Fig. 4:**
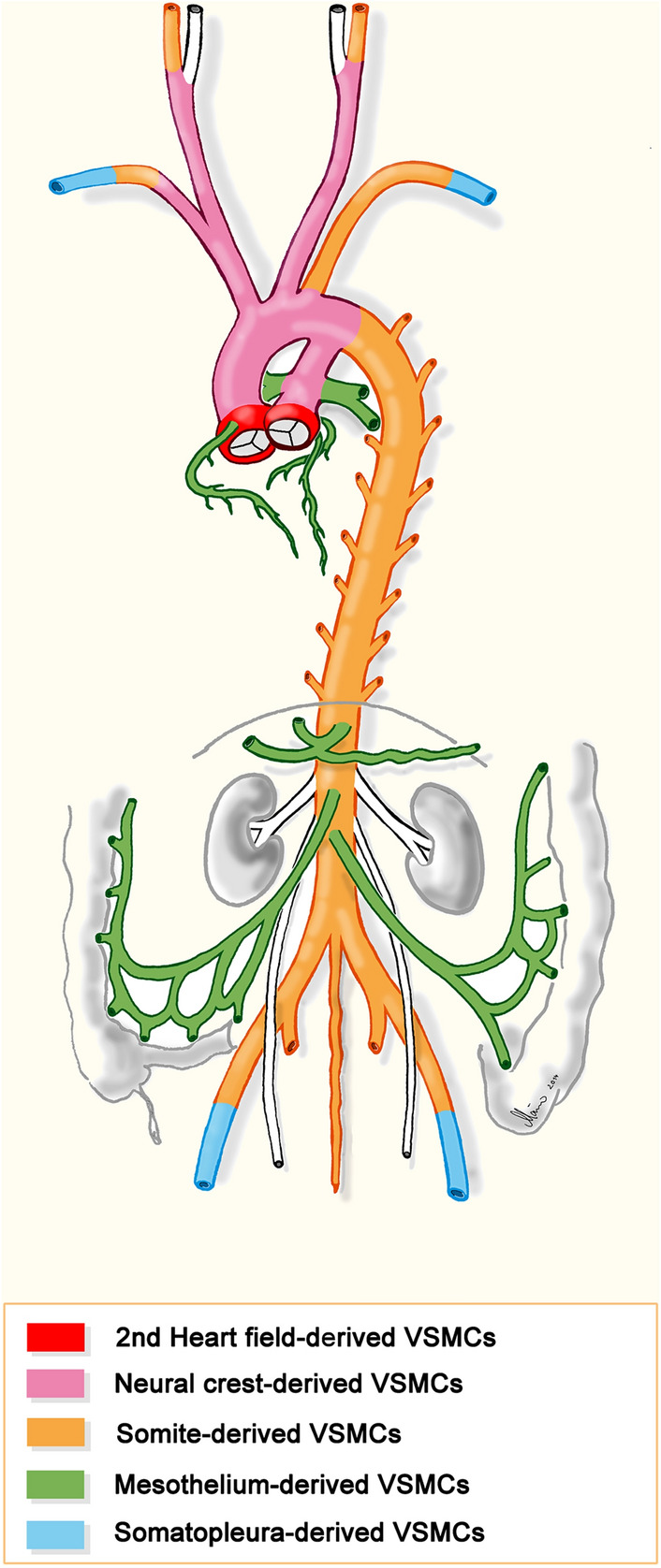
Scheme showing the various embryonic origins of vascular smooth muscle cells forming the tunica media of the aorta and its main branches. (From: Ref. [214]; License Number: 5135340524640)

At electron microscopic level, it can be seen that the smooth muscle cells (SMCs) form an interconnected network [32], and most of them possess an electron-dense dark cytoplasm, while some have a light cytoplasm (Fig. 3C). A functional difference for these cells has not yet been described, however, in mice it was shown that there are two transcriptionally distinct progenitor populations of collector SMCs, NG2-positive and NG2-negative [34]. The embryonic origin of collector SMCs has not been studied in detail. As the collectors usually accompany the arteries, it can be assumed that their SMCs originate from embryonic compartments, comparable to the origin of blood vascular SMCs (Fig. 4).

In contrast to initial lymphatics, LECs of collectors are negative for Lyve-1, Podoplanin (D2-40 antibody) and ESAM-1, but positive for CD31, PROX1, CCBE1, vimentin and β-catenin [32, 35]. Thereby, β-catenin is an important mediator of the WNT-signaling pathway, which has an important role in valve formation in the collectors [36].

**Fig. 5:**
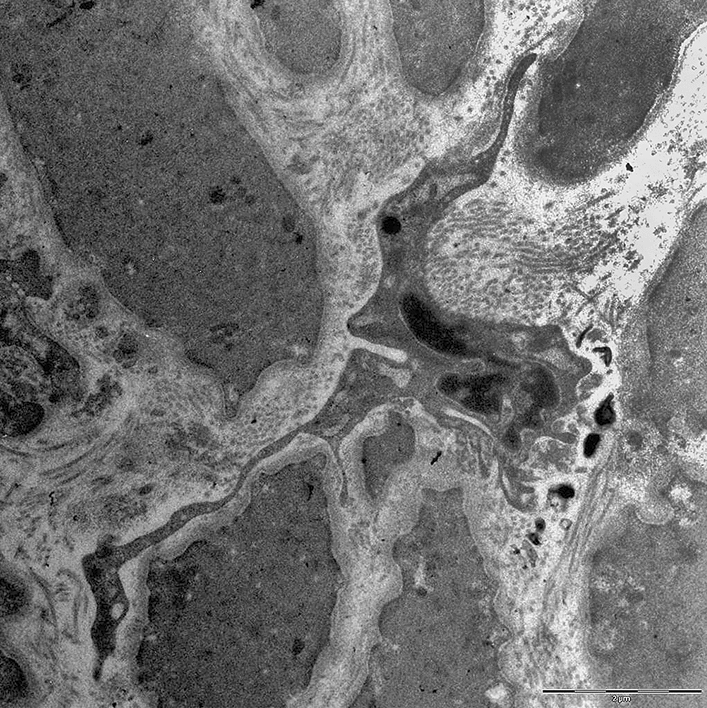
Transmission electron microscopy shows an ICLC with indented nucleus and several slender cellular processes that often reach far between the SMCs. Note, only the initial parts of the processes lie within the section plane. Bar = 2.5 µm

A functionally important cell type in the media of the collectors are the interstitial Cajal-like cells (ICLCs) [37, 38], which appear to be the pace-makers for autonomous contractility of the collectors. They are ramified cells with long slender processes, which come into immediate neighborhood of numerous SMCs (Fig. 5). At the contact sites, the SMCs typically possess multiple caveolae [32]. ICLC possess morphological and molecular congruences with the interstitial cells of Cajal in the gastro-intestinal tract. The cells express Platelet-derived growth factor receptor α (PDGFRα), which, besides DOG1 (Discovered on GIST-1 = Anoctamin 1/ANO1), is a marker for gastro-intestinal stroma tumor (GIST) [39]. It was shown that Ano1 is a major component of the murine lymphatic action potential [40]. Increasing the contractility of lymphatic collectors is an attractive therapeutic approach, and numerous targets have been identified in human collectors [32]. Sympathetic innervation of collectors and stimulating effects of α receptors on pumping have been shown [41]. In addition to norepinephrine, substance P, bradykinin, serotonin, prostaglandin F2a, histamine, and dopamine increase the contraction amplitude or frequency of the collectors. Opposite effects include vasoactive intestinal peptide, atrial natriuretic factor, adenosine triphosphate, (cyclic) adenosine monophosphate, calcitonin gene-related peptide, hydrogen peroxide, oxygen radicals, and low pH [42, 43]. Besides contraction, dilatation of lymphatic collectors is of great functional relevance [44, 45], and lymphangiospasm, which may occur in the course of inflammation, can aggravate edema [23].

## Developmental heterogeneity of somatic and splanchnic lymphatics: clinical significance

Our understanding of the embryonic origin and molecular control of the development of lymphatic endothelial cells (LECs) has expanded considerably. Early development of LECs is controlled by the transcription factor Sox18 in Lyve1-positive cardinal vein endothelial cells [46], which induces expression of the master regulator of LEC development and maintenance, the transcription factor Prox1 [47]. For reviews on embryonic and organ-specific development of LECs see Ref. [48–50]. In mice, LEC proliferation is highest at embryonic day 14.5–16.5 [51], but lymphatics are not completely developed before postnatal day 28 [52]. However, LECs not exclusively arise by transdifferentiation of venous ECs [53–55], but also have a non-venous, mesenchymal origin in pig, turtle, avian, amphibian and murine embryos [56–62]. In the latter, the origin from hemogenic endothelial precursor cells has also been described [63].

**Fig. 6:**
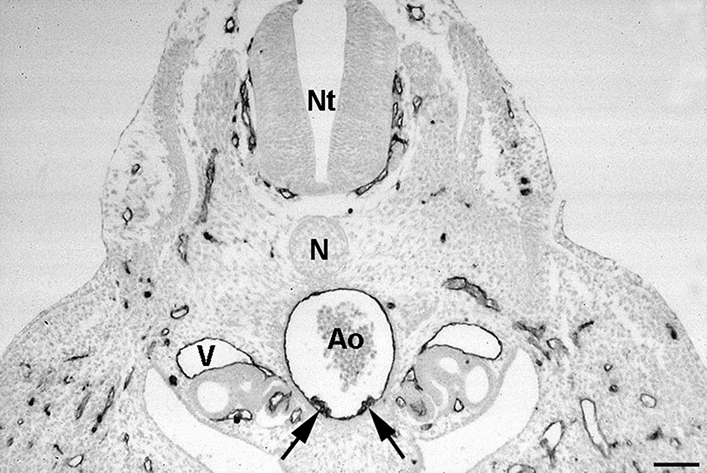
Transvers section of an embryonic-day-4 quail embryo stained with the QH1 antibody (DSHB, Iowa) showing hemogenic endothelium (arrows) in the floor of the dorsal aorta (Ao). N: notochord; Nt: neural tube; V: cardinal vein. Bar = 30 µm

The heterogenous origin of LECs is reminiscent of the heterogenous origin of blood vascular endothelial cells (BECs). BECs develop from (i) angioblasts, which are located in the paraxial and intermediate mesoderm of the embryo from where they migrate into the somatic mesoderm, and from (ii) hemangioblasts (hemogenic and angiogenic) in the extraembryonic and splanchnic mesoderm associated with endoderm [64, 65]. Hence, BECs in the body wall and the viscera have different origins. Hemangioblasts also integrate into the floor of the aorta and vitelline artery, and are then called hemogenic endothelial cells (Fig. 6). The cells give rise to blood cells and hematopoietic cells that will colonize liver, spleen and bone marrow [66].

**Fig. 7:**
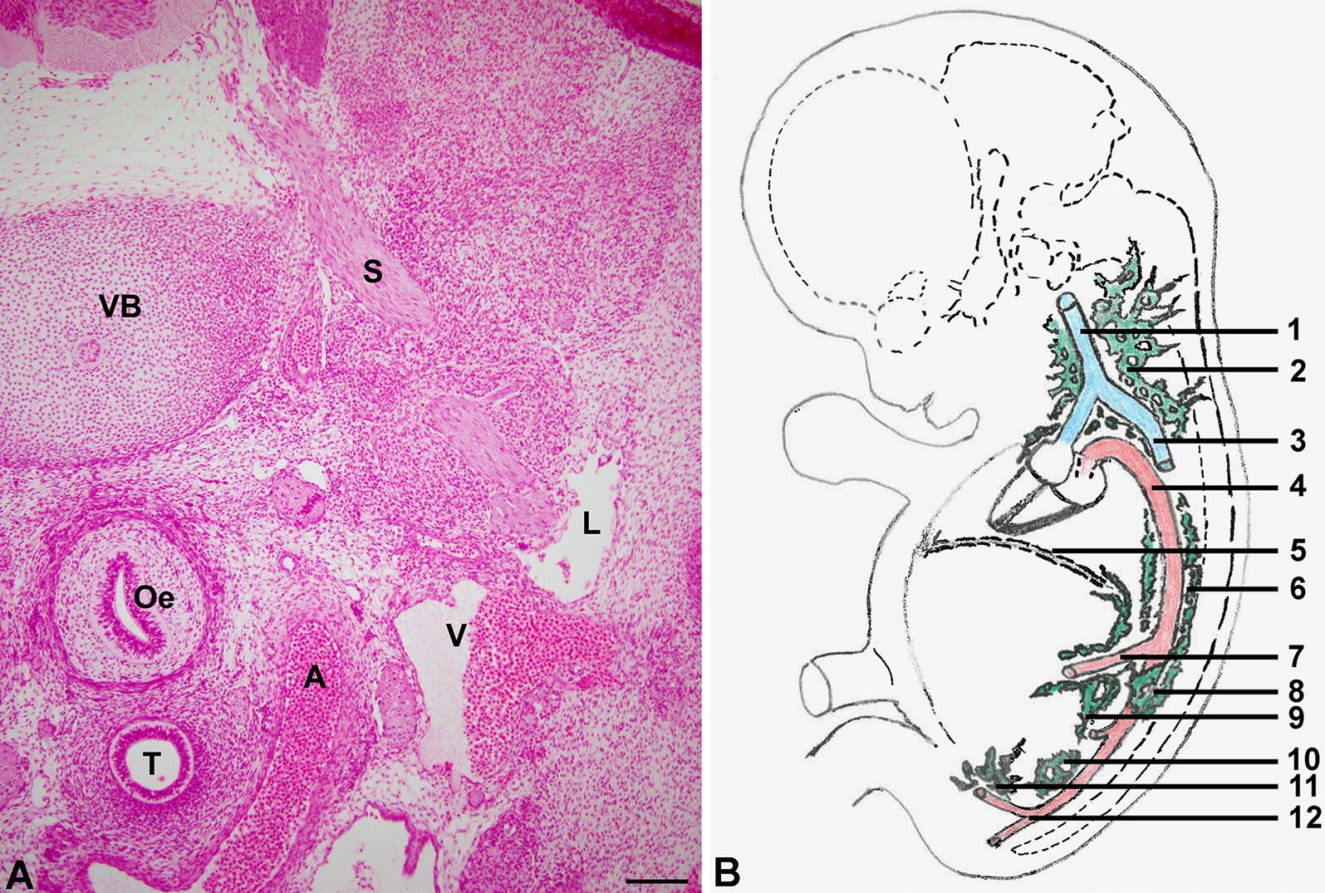
**A** Carnegie stage 18 human embryo (week 7, 13–17 mm CRL). Frontal section showing the jugular lymph sac (L) adjacent to the jugular vein (V) and the carotid artery (A). Also visible are: Oe–oesophagus; S–spinal nerve; T–trachea; VB–vertebral body. We are grateful to Prof. S. Yamada for giving us the opportunity to photograph the specimen at the Kyoto human embryology collection. Bar = 300 µm **B** Schematic of a human embryo corresponding to approx. 30 mm CRL showing the localization of the main anlagen of the lymphatic vascular system. 1: cranial cardinal vein (internal jugular vein); 2: Jugulo-axillary lymphatic plexus (lymph sac); 3: caudal cardinal vein; 4: Aorta; 5: Diaphragm; 6: Thoracic duct (paired); 7: Superior mesenteric artery; 8: Cisterna chyli; 9: Mesenteric (retroperitoneal) lymph sac; 10: Lumbar lymph plexus; 11: Iliac lymph plexus (10 and 11 collectively called posterior lymph sac); 12: Aortic bifurcation. Drawing based on a schematic by B. von Gaudecker; p355 in: [215]

**Fig. 8:**
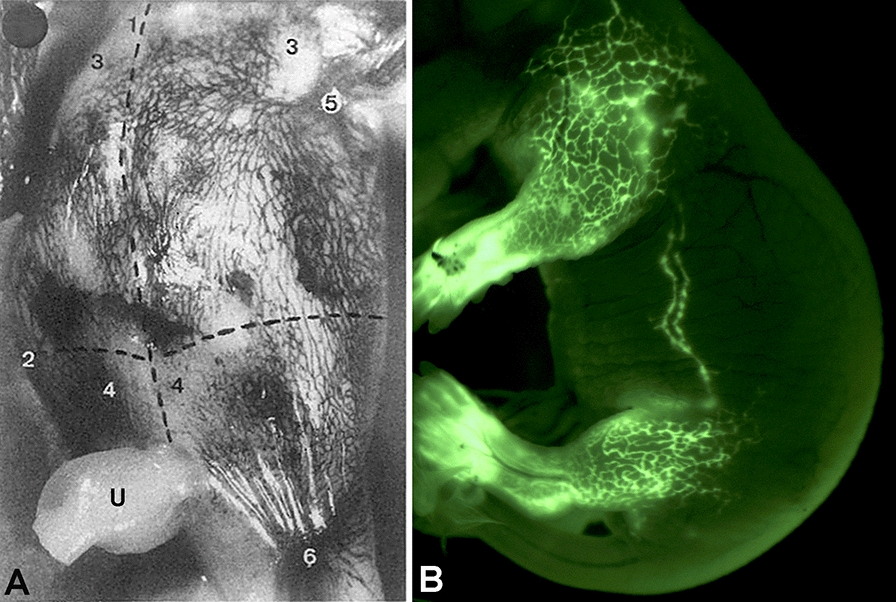
**A** Injection of Indian ink into dermal lymphatics of a human embryo of 70 mm crown-rump length. Numbers ‘3’ and ‘4’ demarcate the upper and lower quadrants of the ventral body wall. ‘5’—axilla; ‘6’—inguinal region. The midline (marked as dotted line ‘1’) is crossed by lymphatics, and this also holds true for the border between thorax and abdomen (marked as dotted line ‘2’). U–umbilicus. (From: [216], with permission of the Gesellschaft Deutschsprachiger Lymphologen). **B** Dermal lymphatic network of a mouse embryo at ED18.5 as demonstrated by injection of 2000 kDa Fitc-dextran into the paws. Note the connection between inguinal and axillary region

**Fig. 9:**
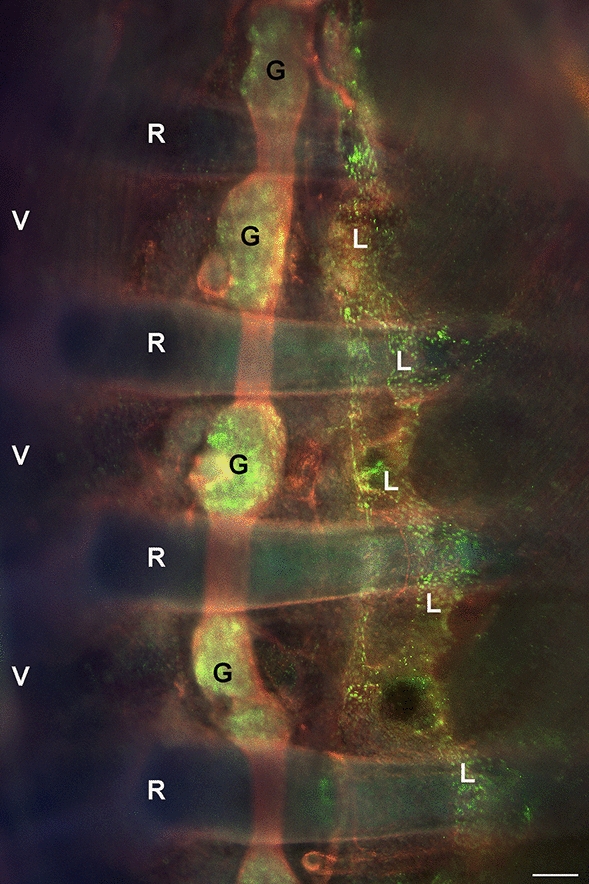
Whole-mount immunofluorescence staining of ED 17.5 mouse embryo with antibodies against neuron-specific β III tubulin (Abcam, 78,078; red) and rabbit-anti-Prox1 (Reliatech, Wolfenbüttel, Germany; green). The sympathetic trunk is marked in red; the nuclei of the ganglia are Prox1-positive (green). Lateral of the sympathetic trunk, a longitudinal lymphatic vessel (L) with Prox1-positive nuclei (green) is visible traversing the ribs (R). V–vertebra. Bar = 500 µm

The first histologically visible anlagen of the lymphatic vascular system are the lymph sacs, especially the largest, the jugulo-axillary lymph sac (Fig. 7A, B). By sprouting of LECs from the lymph sacs and integration of lymphangioblasts lymphatic networks develop and expand. The molecular control of LEC development and specification has been reviewed thoroughly [48, 67]. In the skin of mice, the number of lymphangioblastic integration is much larger in the lumbo-sacral region as compared to the cervico-thoracic region [62]. This may correspond to the observation that primary lymphedema in humans (and even in mice) is more often localized in the legs (hindlegs of mice) as compared to the arms where secondary, breast cancer-related, lymphedema is dominant [68–70]. Despite the heterogenous origin, a continuous dermal lymphatic network develops which connects the inguinal and axillary regions (Fig. 8A). This is somewhat at odds with the general observation that thoracic lymph drains axillary and abdominal lymph drains inguinal, and a so-called watershed is located at the level of the costal arch. But the watersheds are never absolute and with manual lymphatic drainage one is always able to shift interstitial fluid from one tributary area to another. In mice, superficial lymphatic collectors exist which connect inguinal and axillary regions (Fig. 8B) [68]. This explains why tumors injected subcutaneously into the flank of mice rapidly metastasize to axillary lymph nodes. In mice, additional longitudinal lymphatics are found accompanying the sympathetic trunk (Fig. 9). The existence of such paravertebral lymphatics may explain why the blockage/ligation of the thoracic duct in humans is well tolerated and does not necessarily produce LE [71].

The visceral lymphatics also have a dual origin by transdifferentiation from embryonic veins and from mesenchymal cells which were characterized in part as hemogenic endothelial precursors [63]. These authors show that in mice the retroperitoneal (mesenteric) lymph sac (no. 9 in Fig. 7B) develops from the mesenteric vein. Most of the intestinal lymphatics then develop from mesenchymal cell clusters that coalesce with the mesenteric lymph sac. Development of intestinal lymphatics is highly dependent on VEGFR-3/PIK3CA signaling. In double heterozygous mutants, intestinal lymphatics were almost absent while dermal lymphatics were normal. Additionally, lymphatics of the diaphragm, which is part of the body wall, developed normally, revealing a clear difference between somatic and splanchnic lymphatics as concerns the quantitative dependence on the VEGFR-3 signaling cascade [63]. Accordingly, Chy-mice, which carry a deletion of the *VEGF-C* gene, develop chylous ascites, and lymphedema in the hind paws [68]. In the past years, a number of molecular differences between somatic and splanchnic lymphatics have been described. The knock-out of the wingless-type MMTV integration site (WNT) family member Wnt5a induces lymphatic cyst formation in the dermis, while intestinal lymphatics are normal [51]. In contrast, postnatal deletion of VE-cadherin destroys intestinal lymphatic vessel integrity, but not dermal [72].

**Fig. 10:**
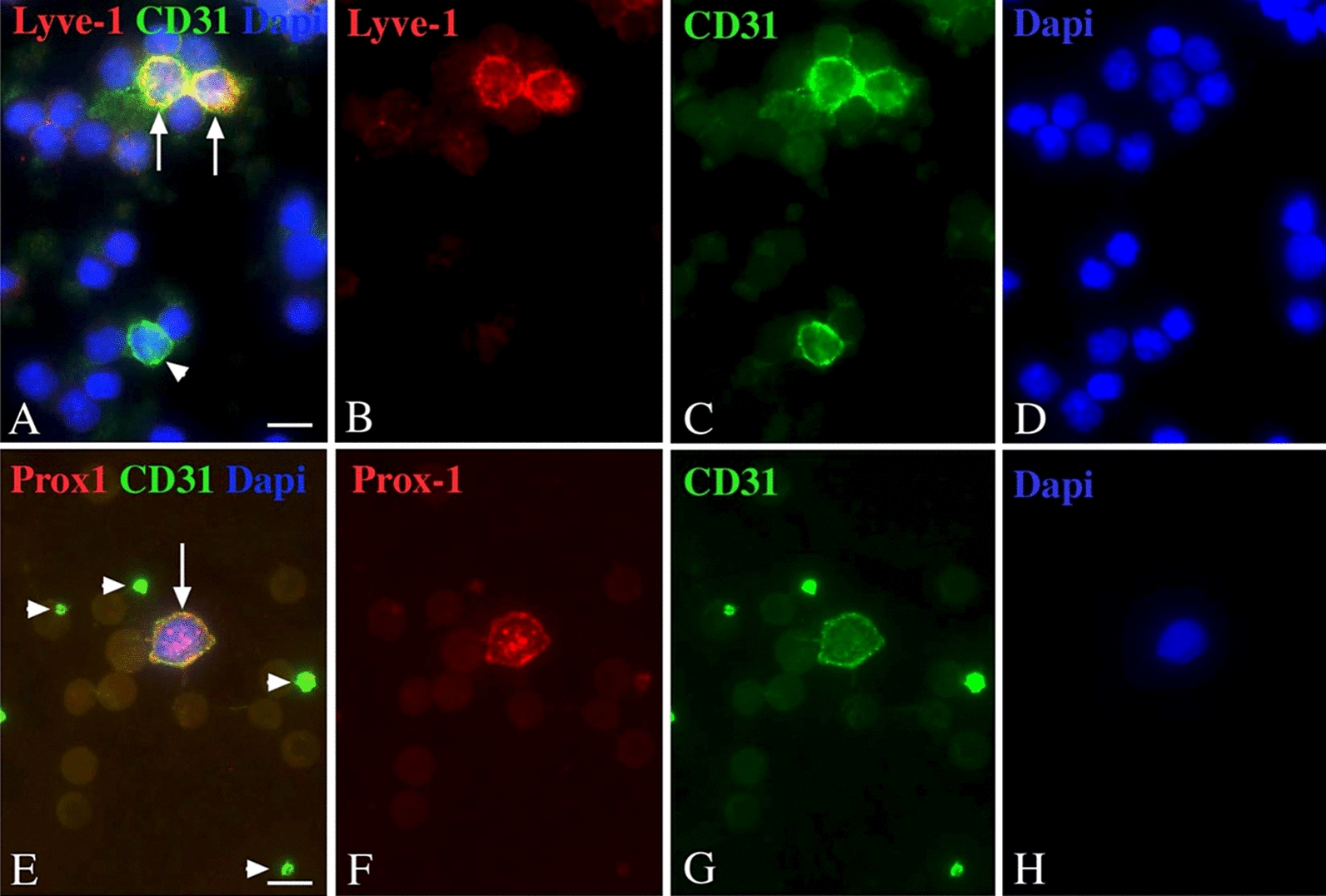
Immunocytology of pediatric blood stained with antibodies against pan-endothelial marker CD31(green) and lymphendothelial markers LYVE-1(A-D) and PROX1 (E–H) (red). Nuclei are stained with DAPI. Note double-positive cells (arrows). Platelets and some cells are only CD31 positive (arrowheads). Bar = 10 µm. Data and photograph courtesy by Dr. K. Buttler, Göttingen

The complexity of finely distributed anlagen of the lymphatic vascular system has also been observed in human embryos [73]. It is very probable that both transdifferentiation of venous ECs and integration of lymphangioblasts contribute to lymphangiogenesis in human embryos, and there is evidence that lymphangioblasts are still present in the adult. There, lymphangioblasts even seem to have the potential to circulate in the bloodstream. Renal transplant rejection is often accompanied by inflammation and lymphangiogenesis. Recipient-derived LECs in newly formed lymphatics in transplanted kidneys point to the existence of a highly mobile progenitor cell [74]. Furthermore, in renal transplant patients who developed post-transplant Kaposi sarcoma (KS), Barozzi and coworkers [75] observed that more than 70% of the tumor mass (both endothelial and spindle cells) harbored markers of the donors. KS is a multi-focal tumor of the skin and the mucous membranes, and the data point to a highly mobile, circulating cell-of-origin with lymphendothelial characteristics. In pediatric blood, we found middle-sized lymphocytes co-expressing the LEC markers PROX1 or LYVE1 with the pan-endothelial marker CD31 (Fig. 10). We calculated that pediatric blood contains an average of 117 LYVE1/CD31^+^ cells per µl and 35 PROX1/CD31^+^ cells per µl.

## Functions of the lymphatic vascular system

### Fluid homeostasis

The most obvious function for lymphatics is fluid homeostasis, the return of fluid extravasated from the blood vessels back into the blood stream. Differences between intra- and extravascular hydrostatic and colloid-osmotic pressures are important parameters that were already considered by Starling [76]. Starling described that he did not find any evidence for the uptake of edema fluid from limb connective tissue into the blood vessels, and he clearly pointed out to the function of lymphatics in this respect. Later, Starling described the filtration of blood in the arterial part of the capillaries and reabsorption in the venous part, which was included into textbooks as Starling’s equilibrium until a couple of years ago [77]. It has then become evident that the interstitial oncotic pressure and the endothelial glycocalyx are important factors, and blood vessels are much less permeable than usually calculated [78, 79]. In addition, pericytes, which are present on capillaries and postcapillary venules, are important regulators of vascular integrity [80]. Therefore, the filtration rate is much lower than originally calculated and the filtrate is quantitatively taken up by lymphatics.

Under physiological conditions, interstitial fluid is taken up by blood capillaries only in kidneys, intestine and lymph nodes. The latter is important for reabsorption of lymph into the blood. It has been calculated that the daily capillary filtration rate in a 70 kg human is about 5 L. Of these, approx. 50% are reabsorbed in lymph nodes, due to the fact that the oncotic pressure of blood is much higher than the oncotic pressure of lymph, which contains only half the protein of serum [78, 81]. The blood vessels of the lymph nodes are constantly flushed with fresh lymph, and it is generally believed that humans have a particularly large number of lymph nodes. In fact, the daily inflow of lymph via the thoracic duct was reported to be 1–3 L [78, 82, 83]. The amount of lymph transported by the right lymphatic duct is not known in humans. In dogs it was shown to be only 5% of the thoracic duct lymph [84]. The amount of lymph measured in small animals cannot be extrapolated to humans. In rats and mice, 55.6 and 26.3 ml/day, respectively, were measured by thoracic duct cannulation [85], which would correspond to approx. 13 or 68 L per day for a 70 kg person.

### Hyaluronan turnover

Lymphatics are of utmost importance for the turnover of hyaluronan (HA). HA is a non-sulfated linear glycosaminoglycan of 250–50.000 disaccharides consisting of D-glucuronic acid and N-acetyl-D-glucosamine. It is produced by HA synthases 1–3 (HAS1-3) at the cytoplasmic side of the cell membrane of numerous cell types, and is extruded through pore-like structures [86]. The water-binding capacity of HA is extremely high (1 g can bind 6 L of water, and thereby produce a gel), and its turn-over, especially in the skin, is within hours [87]. HAS1 is most highly expressed in smooth muscles cells and fibroblasts, as well as keratinocytes and melanocytes (https://www.proteinatlas.org/ENSG00000105509-HAS1/celltype). RNA expression of HAS2 and HAS3 are very similar; however, HAS2 is additionally found in glandular cells and (blood vascular) endothelial cells. With RNASeq of three different LEC isolates, we found expression of HAS1-3 only at extremely low, almost neglectable levels (unpublished data).

HA binds the leukocyte receptor CD44 and the CD44-homolog receptor LYVE-1 [88]. LYVE-1 is highly expressed on both the luminal and abluminal membrane of LECs from initial lymphatics [89]. It mediates internalization and degradation of HA, its release into afferent lymph for further degradation in lymph nodes. Only 10% of HA remain in efferent lymph and are finally degraded in LYVE-1-positiv liver sinusoids [87]. Thereby, LYVE-1 may interact with the HA scavenger receptor HARE (also termed Stabilin-2), which we found highly expressed in LECs by microarray and RNASeq analyses (unpublished data). Of the three hyaluronidases (HYAL 1–3) which degrade HA, we found highest expression of HYAL2 in LECs, which remains constant under hypoxia (unpublished data). The importance of HA degradation and removal becomes immediately obvious in lymphedema (LE), which is a chronic disease evoked by insufficiency of the lymphatic vascular system. In stage-1 LE, accumulation of HA has been found [90], giving rise to a deeply impressible (pitting) edema (*AWMF Online Portal Wiss. Med. Registry no. 058–001; valid until 15.04.2024*). In aesthetic medicine, HA-based dermal fillers are in use. In the event of a vascular occlusion that may result from an accidental intravascular injection, hyaluronidase can be administered [91]. Treatment of LE with hyaluronidase has only rarely been reported but could be promising [92]. In stages-2 and -3 LE, the interstitium becomes increasingly fibrosclerotic, and adipose tissue develops. The reasons for these changes are unknown, although, hypoxia may be a driving factor [93, 94]. The body's attempt to store the high caloric HA in fat cells could be a reason for adipose tissue development. In sum, the lymphatics are of greatest importance for removal of HA, and their malfunction results in a chronic, progressive LE.

### Cerebrospinal fluid (CSF) uptake

As in the skin, HA is also a major component of the intercellular spaces of the central nervous system (CNS) [95]. Although these spaces are on average only about 40 nm wide, they account for about 20% of the brain volume. Thereby, the CNS consisting of the brain and spinal cord, is directly connected to the peripheral nervous system (PNS). Both, CNS and PNS are mostly regarded as immunologically privileged organs, protected by the blood–brain barrier (BBB) and blood-nerve barrier (BNB). The morphological correlate of these barriers are the blood vascular endothelial cells (exception: the circumventricular organs, see: [96]) and the neurothelium at the dura-arachnoidea interface and the endoneurium-perineurium interface [97]. Usually regarded as immune privileged organs, CNS and PNS do not contain lymphatics, although they possess an organ-specific immune system [98, 99]. Nevertheless, blockage of cervical lymphatics induces brain edema [100]. The CNS has a communicating ventricular system with (traditionally) approx. 150 mL of CSF (35 mL in the ventricular system and 115 mL in the subarachnoid space). However, recent MRI measurements showed values in the range of 250–330 mL [101]. Replacement of fluid occurs at least 3 times per day [102]. Virtually all studies of CSF outflow tracts conclude that the markers used, or dendritic cells, are found after a short time in superficial and predominantly the deep lymph nodes of the neck [103–106]. The markers were also detected in many other lymph nodes, mainly in lumbar region [107]. Lymphatics are directly attached to the dura mater and the perineurium of cranial and spinal nerves [108]. They are found at specific locations at the base of the skull, and also run in parallel to meningeal arteries and venous blood conduits [52, 109, 110]. Similarly, along the optic nerve CSF reaches the uvea of the eye and diffuses through the sclera to the conjunctival lymphatics [111]. Cranial CSF outflow is significantly higher than spinal [112]. CSF diffuses through dura, sclera, and perineurium to be taken up by lymphatics. The significance of the CSF-lymph communication in health and disease was noted very early and is currently being discussed again intensively, e.g. in relation to neuroinflammatory diseases and aging [101, 109, 113, 114].

### Immune surveillance

The special importance of the lymphatic vessels for immune surveillance becomes clear when one considers that the body regions affected by lymphedema have a very high susceptibility to fungal infections and erysipelas. Erysipelas (called cellulitis in the USA) is a painful infection caused by group-A streptococci, which is usually treated with antibiotics [115]. But, as we discuss below, lymphatics are a double-edged sword. They can both promote inflammation but also resolve it. For the latter, high amounts of lactoferrin are present in normal lymph [116]. Due to its enormous iron-binding capacity, lactoferrin is a potent anti-bacterial agent with additional antiviral effects [117, 118]. Besides erysipelas, an involvement of lymphatics in immune and autoimmune diseases has been describe for: lymphangitis, cat-scratch disease, filariasis, inflammatory bowl diseases including ulcerative colitis and Crohn’s disease, rheumatoid arthritis, psoriasis, chronic venous insufficiency, Diabetes mellitus, atherosclerosis and others [50, 119, 120].

Communication of tissues with the immune system is managed to a significant extent by lymphatic vessels. Leukocytes usually immigrate into tissues by diapedesis through capillaries and postcapillary venules, and emigrate via the lymphatics, when present. While afferent lymph has only a small proportion of leukocytes, this is significantly higher in the efferent lymph that has passed the lymph node. Leukocytes in efferent lymph are: 80% T-lymphocytes, 6–10% dendritic cells, 1–4% B-lymphocytes, and 2–8% monocytes [121]. Lymphatics are not present in the cornea, due to high expression of inhibitors of both hemangiogenesis and lymphangiogenesis [122, 123], but may develop after corneal transplantation, and are then a major determinant of transplant rejection [124].

The attraction of leukocytes by LECs is mediated by diffusible factors; e.g. attraction of dendritic cells is mediated by the chemokine CCL21 via its receptor CCR7. Transmigration of neutrophils is regulated via CXCL8 (IL8) on LECs and its receptors CXCR1/2 on neutrophils [121, 125–127]. The immunoregulatory T_REG_ cells employ the tumor necrosis factor (TNF) superfamily member lymphotoxin (LT) to enter afferent lymphatics [128]. Otherwise, LT is well-known for its potential to organize the development of lymphoid organs [129]. During inflammation, the numbers of dendritic cells and T cells in lymph increase significantly [130, 131].

The chemotactic lipid sphingosine-1-phosphate (S1P) is found in all body fluids and with highest concentration in lymph. S1P mediates the exit of lymphocytes from lymph nodes into efferent lymphatics [132, 133]. S1P is a phosphoric-acid esters of sphingosine, a monounsaturated amino alcohol of 18 carbon atoms. Sphingosine forms the backbone of sphingolipids. However, S1P is a lipid mediator with multiple intra- and intercellular functions [134]. It can induce cyclooxygenase 2 (COX2) expression and prostaglandin E2 (PGE2) secretion, thereby linking S1P to inflammation, vascular hyperpermeability and pain sensitization [135]. S1P is elevated in obesity [136], which may render obese patients even more susceptible to inflammation in LE. In mice, lymph-targeted inhibition of COX-2 could reverse mesenteric lymphatic dysfunction, visceral obesity and inflammation [137].

As already noted, lymphatics may promote inflammation by acting as pathways for dendritic cells towards the lymph nodes; e.g. in organ transplantation [124, 138]. Thereby, inflammation induces lymphangiogenesis and lymphatic vessel density via chemokines and cytokines such as IL1, TNFα and CCL2. On the other hand, lymphatics resolve inflammation e.g. by expression of the chemokine scavenging receptor ACKR2, which is also known as chemokine receptor D6 or CCBP2 [139–141]. ACKR2 is capable of binding, internalizing and scavenging the majority of CC-chemokines [142]. We studied three foreskin-derived human, dermal LEC isolates under normoxia (21% pO2) and hypoxia (1% pO2). With RNASeq, we observe statistically significant upregulation of ACKR2 under hypoxia [143]. This seems counterintuitive to the observation that inflammation (erysipelas) is enhanced in hypoxic LE tissue. However, ACKR2 is a lowly expressed gene. Also, the sum of pro- and anti-inflammatory mechanisms must be considered, and the ability of leukocytes to freely circulate.

**Fig. 11:**
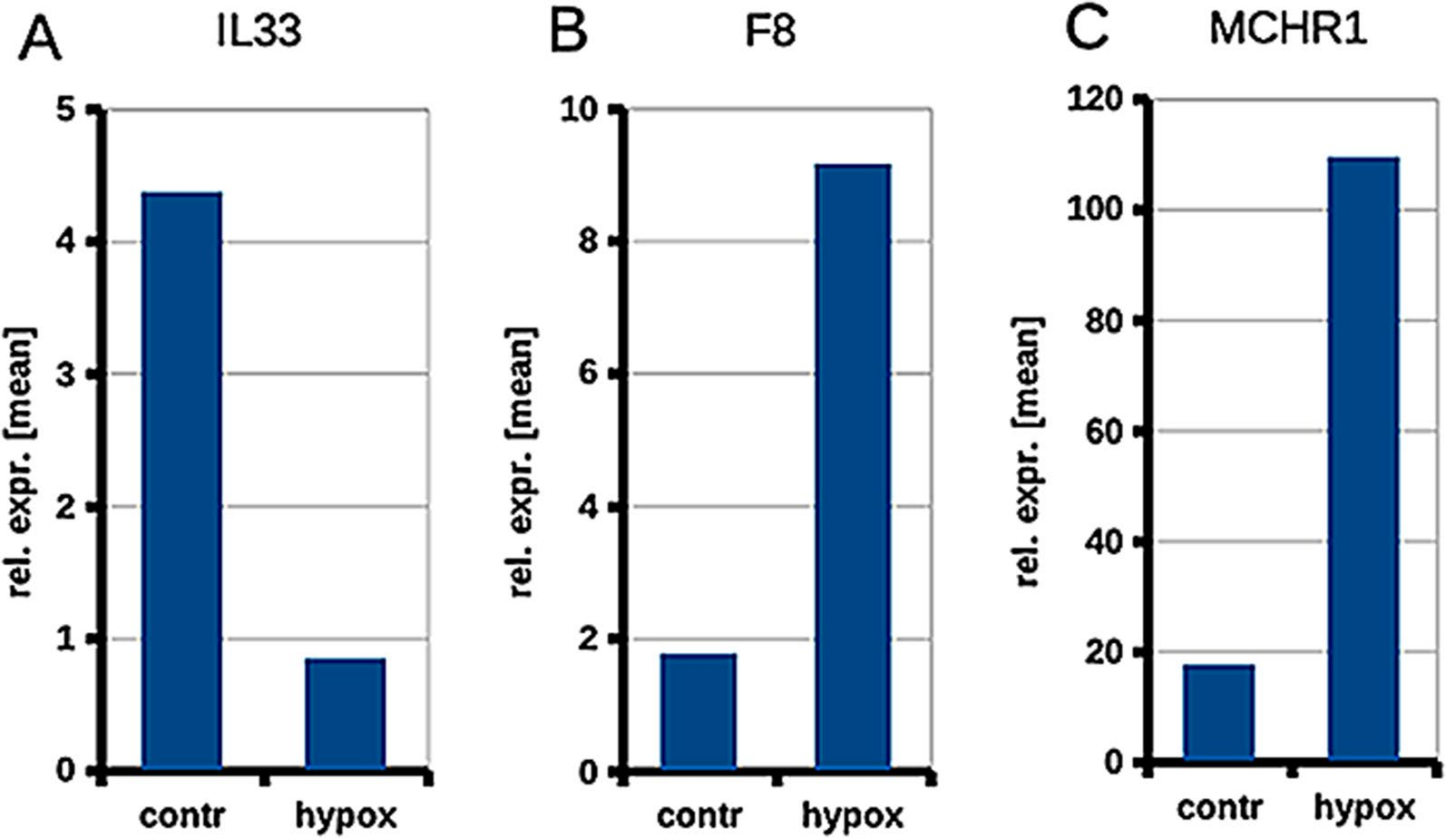
Real-time PCR of LECs under normoxia (contr) vs. 1% pO_2_ (hypox). Mean values of 3 LEC isolates and each 3 replicates are shown for **A** IL33; **B** F8; and **C** MCHR1. Values were calculated with the δδCT method. Beta-actin was used as a housekeeper. Note significant down-regulation of IL33, and upregulation of F8 and MCHR1 by hypoxia

**Fig. 12:**
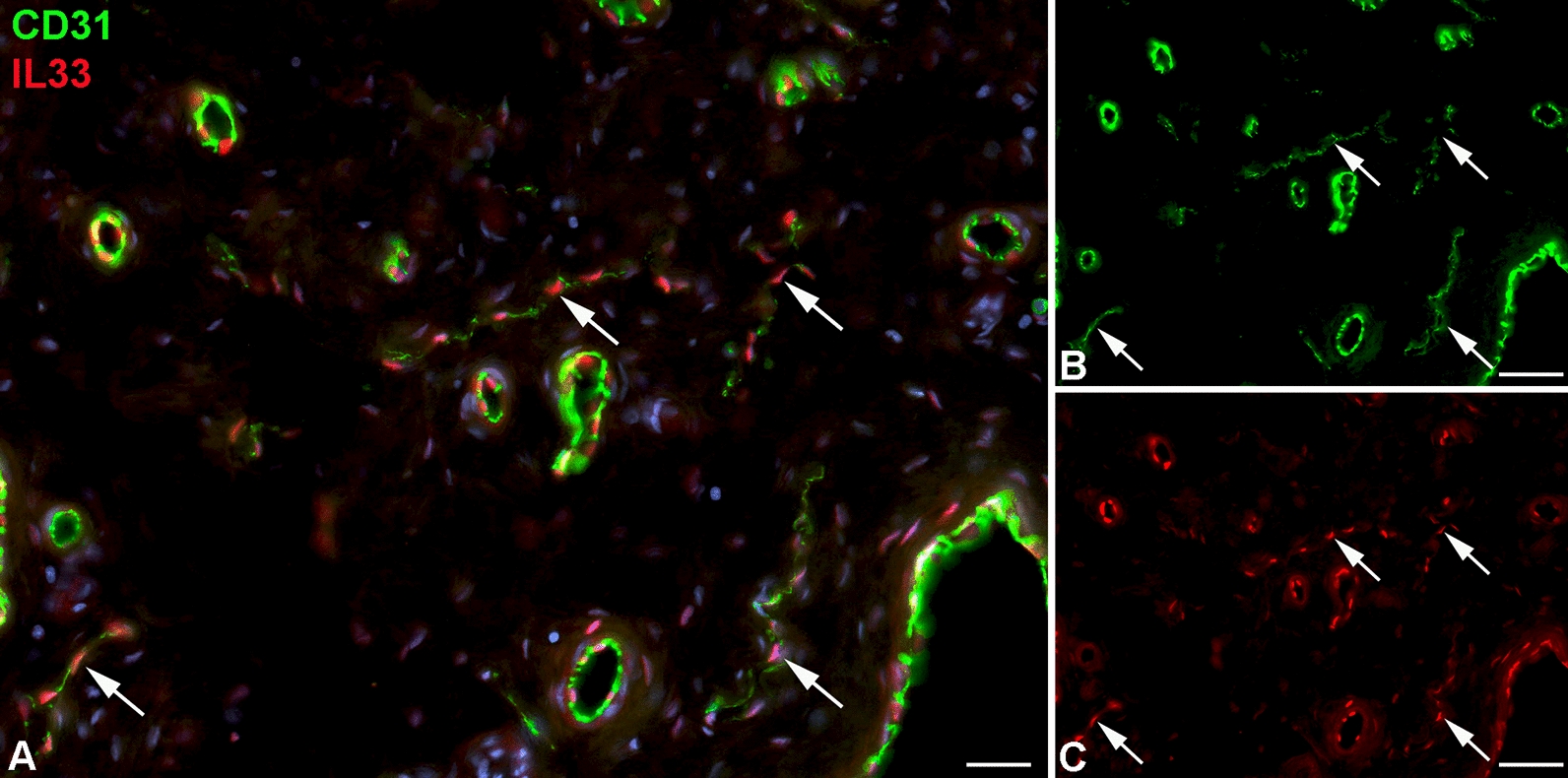
Immunofluorescence studies of foreskin tissue with antibodies against CD31 (green) and IL33 (red). **A** Merged picture of (**B** and **C**). BECs are strongly positive for CD31, initial lymphatics (arrows) possess week expression. IL33 is found in the nuclei of both BECs and LECs. Bar = 35 µm in (**A**), and 70 µm in (**B**, **C**)

In contrast to ACKR2, we observed significant down-regulation of interleukin 33 (IL33) under hypoxia (Fig. 11A; [143]). Immunofluorescence studies of foreskin show expression of IL33 in both BECs and LECs (Fig. 12). IL33 has been described as an interleukin typically expressed by blood vascular endothelial cells (BECs). It is stored as a nuclear precursor and released upon cell damage. It can attract T helper-2 (Th2) cells, which are important for an isotype-switch of B cells, and react as an ‘alarmin’ [144]. Thereby, it amplifies and improves immune responses [145]. Down-regulation of IL33 may contribute to the decrease of immune surveillance in lymphedematous tissues.

In addition to regulating leukocyte migration, LECs can modulate immune cell activation by antigen presentation [146, 147]. Thereby, their main functions seem to reside in the induction of T cell tolerance and suppression of dendritic cell-mediated T cell activation. One of the main factors for inhibition of immune responses by LECs is the transmembrane protein Programmed cell death-1 ligand-1 (PD-L1, CD274) [148]. Binding of PD-L1 to its receptor PD1 on monocytes induces expression of interleukin 10 (IL10), which limits defense processes and protects the body from self-destruction [149, 150].

### Uptake of chylomicrons

Intestinal villi possess a central lymphatic vessel, which is of utmost importance for the uptake of chylomicrons released at the basal side of enterocytes of the small intestine. Due to the postprandial milky appearance of the lymphatics of the gastrointestinal (GI) tract they were named chylous vessels or lacteals. In 1628, it was Gaspare Aselli [1] who recognized the milky appearance of GI lymphatics. While small and medium chain fatty acids diffuse freely into enterocytes, long-chain fatty acids must be packaged in bile salt-containing micelles, and are then taken up by fatty acid transport proteins. They are re-esterified and secreted basally as chylomicrons up to 1200 nm in size by exocytosis [151]. Uptake of chylomicrons by lacteals is mainly via their microvalves and to some extent by transcytosis [152, 153]. Malformations (primary genetic) of lacteals, or secondary malfunction of these vessels, cause a cascade of tissue changes finally resulting in protein-losing enteropathy [2, 154]. Thereby, lymphostasis can give rise to lymphangiectasia in intestinal villi, chylo-enteral fistula and the loss of chylomicrons, fat-soluble vitamins and lymphocytes into the intestinal lumen. The increase in interstitial pressure leads to the accumulation of proteinaceous fluid in the mucosa and to loss of proteins such as gamma globulins into the intestinal lumen. The invasion of intestinal bacteria into the fistulas combined with immunodeficiency is life-threatening [100]. Medium-chain triglyceride diet was found to be helpful in this condition [155]. While in the human the non-functionality of lacteals is a severe, potentially life-threatening condition, in the mouse, however, protection against obesity has been observed by tightening of the initial lacteals, making them non-functional for the uptake of chylomicrons [153, 156].

### Lymphatics and coagulation

Concentration and activity of coagulation factors V, VII, VIII, IX and X, are significantly lower in lymph as compared to plasma [157]. Nevertheless, in chronic LE partial or complete intravasal coagulation in dilated initial lymphatics and precollectors can regularly be found [158], but the causal relationship between coagulation and lymphedema is unclear [159]. The coagulation potential of lymph had already been noted in 1653 by Olof Rudbeck [17] (cited from: Ref. [23]. High amounts of fibrinogen were detected by proteomic analysis of lymph [116].

Using RNA-Seq of three human LEC isolates, we found high expression of factor VIII (F8, antihemophilic globulin A) and its carrier protein von-Willebrand factor (VWF). Expression of VWF in LECs in vitro has been found previously [160, 161]. Besides liver, LECs were identified as the main source for F8 [162]. Under hypoxia (1% pO_2_), we observed a significant increase in the expression of F8 in LECs (Fig. 11B; [143]), suggesting that tissue hypoxia in LE may have a similar effect and may accelerate the blood clotting cascade.

### Salt balance

Sodium (Na^+^) is present in high concentrations in the extracellular space and is closely coupled to the body’s water balance under control of the kidneys. High dietary sodium chloride intake can induce a fluid shift from the interstitial to the intravascular space [163]. However, besides the mineralized bone, skin is an organ which serves as a reservoir for osmotically inactive sodium [164]. Thereby, GAGs regulate cation exchange under varying dietary conditions and during ageing [165]. Tonicity sensing in skin is performed by cells of the mononuclear phagocyte system (MPS) [166]. By production of Vascular Endothelial Growth Factor-C (VEGF-C) the cells interact with the lymphatics, which, under very high salt diet, may even result in lymphangiogenesis [167]. However, VEGF-C is not only involved in growth of lymphatics but also in maintenance and function [156]. Removal of VEGF-C or blocking of its receptor increases interstitial Cl^−^ accumulation and skin volume. This indicates a function of lymphatics for electrolyte clearance in the skin [167, 168].

In the human, it may also be hypothesized that increased skin sodium levels without volume changes could be a cause of the feeling of tightness described by stage-0 lymphedema patients (without measurable volume increase). VEGF-C expression has not been measured in these patients. Since a couple of years, ^3^Na MRI allows the non-invasive quantification of tissue sodium content [169]. Elevated sodium levels in skin and adipose tissue of patients suffering from lipedema (a disorder of adipose tissue, characterized by symmetric and bilateral enlargement of the lower extremities due to abnormal deposition of subcutaneous fat) may indicate a similar mechanism for increase in tissue tension and touch pain [170].

Thus, the lymphatic vessels of the skin, and possibly in other organs, take on a kind of renal function. This function is reminiscent of the proposed functions of the ‘secondary vascular system’ (SVS) in fish. The SVS possesses multiple small caliber connections to the arteries and veins of the primary vascular system (PVS), but is usually free of erythrocytes [171]. Of note, its endothelial cells express markers found in mammalian lymphatics [172]. However, upon hypoxia or exercise, the diameter of the connections to the PVS increases and allows the passage of erythrocytes. Thereby, the SVS becomes a functional blood vascular system [173, 174]. The function of the SVS is not completely understood. As it forms capillary networks in the gills, mouth and skin it may be involved in respiration, but also ionic and osmotic buffering, suggesting renal functions [174–176].

In the human and mammalian kidney, an osmoregulatory function of lymphatics has not been found yet, although LECs express mineralocorticoid receptor (MR, NR3C2) [177], which in the kidney is of utmost importance for sodium and water reabsorption. In the kidney, lymphatics accompany the major arteries and drain perivascular interstitial fluid. Most parts of the cortex and the medulla do not possess lymphatic vessels [178]. Lymphatics can also be found at capsular and subcapsular positions, and along the pelvis and ureter. In kidney transplant recipients, development of a lymphocele has been observed with a frequency between 1 and 26% [179]. A tense lymphocele can severely affect transplant function. However, due to high regenerative potency of lymphatics, lymphatic congestion usually resolves spontaneously [180].

## Phylogeny of the lymphatic vascular system

The lymphatic molecular character of the secondary vascular system (SVS) of fish raises the question of the phylogenetic significance of the lymphatic system. It is very obvious that during embryonic development the blood vascular system develops first. The cardiovascular system is generally regarded as the first functioning organ system [181], with a focus on its nutritive function. However, immune defenses had to develop early on, in organisms that did not yet require a nutritive circulation system due to their small size. Accordingly, macrophages, as part the innate immune system, are present in earliest stages of embryonic development, even before gastrulation, suggesting that immune functions are more ancestral than oxygen transport functions. This is supported by the fact that insects, which possess a dorsal heart but not yet endothelial cells, circulate immune cells and interstitial fluid (lymph) through their body [182]. Oxygen transporting molecules comparable to hemoglobin (arylphorin, hemocyanin) are present in various insects, arthropods and mollusks [183], but these molecules still have immune functions. In crustaceans, infection induces the cleavage of hemocyanin into antibiotic peptides [184]. In cephalopods, hemocyanin binds to various particles and seems to act like an opsonizing agent that increases the activity of phagocytes [185].

The most important growth factor receptor for LECs, Vascular Endothelial Growth Factor Receptor-3 (VEGFR-3), is expressed in early embryonic blood vessels before it becomes restricted to the lymphatics, and is essential for early blood vascular development [186]. The lymphangiogenic members of the VEGF [187, 188] family, VEGF-D and VEGF-C, have evolved much earlier than the hemangiogenic member VEGF-A. From this, there is much evidence that the lymphatic vascular system has more primal functions than the blood vascular system. In fish, the lymphatic-like SVS still has substitute blood vascular functions, and during embryonic development but also during regeneration of the anal fin of adult fish, it is capable of transdifferentiating into a *bona fide* blood vascular system [189].

In higher vertebrates, the original lymphatic vascular system may have transformed into a VEGF-A-dependent blood vascular system and the lymphatic vascular system is formed again by recourse to VEGF-C-dependent mechanisms. As discussed above, the mechanisms by which the lymphatics are formed are largely comparable to those of blood vessels. The biogenetic law of Ernst Haeckel [190], according to which ontogenesis is a recapitulation of phylogenesis, may well be doubted with regard to the development of the vascular systems [191].

## Lymph and the development of adipose tissue

In stage-II and -III LE, massive accumulation of adipose tissue is highly characteristic of the disease, and gave rise to the statement ‘lymph makes you fat’ [50, 58, 192, 193]. The underlying mechanisms are not known. The most likely causes are accumulation of adipogenic factors or direct provision of high-caloric substances by the lymph.

Preadipocytes are present in connective tissue throughout life, and the most potent factors that control differentiation of preadipocytes into adipocytes are Peroxisome proliferator-activated receptor gamma (PPARγ) and CCAAT-enhancer-binding protein alpha (C/EBPα). Insulin-like growth factor 1 (IGF-1) signaling induces preadipocyte differentiation whereas wingless-type MMTV integration site family members (WNTs) are regarded as negative regulators of adipogenesis [194].

As discussed above, lymphatic vessels are paramount for HA clearance. Accumulation of HA in LE might be a trigger for adipogenesis, as it is a highly caloric molecule that the body will put to further use. The high amount of S1P in lymph could act in a similar way. Physiologically, the primary site for S1P clearance is the liver [195]. The development of adipose tissue is to a large extent dependent on caloric intake. For *Drosophila melanogaster* it was shown that the S1P-to-ceramide ratio is a regulator of postprandial satiety and caloric intake-independent obesity [196]. A potential systemic function of the lymphatics on appetite regulation will be discussed below.

In the same line, leakage of chylomicrons from intestinal lymphatics (lacteals), as shown in mice with endothelial-specific deletion of the transcription factor *Prox1*, induces obesity [193]. In vitro, application of mesenteric lymph or chylomicrons to the medium greatly enhances adipogenesis [193, 197, 198]. Our studies on the effects of hypoxia (a hallmark of LE) on LECs show significant up-regulation of Angiopoietin-like 4 (ANGPL4) [143], a secreted inhibitor of lipoprotein lipase (LPL). LPL is produced and secreted by adipocytes and cardio/myocytes and hydrolyses triglycerides (TGs), which are carried in blood and body fluids as very low-density lipoproteins and chylomicrons [199, 200]. ANGPTL4 increases TG levels while loss-of-function mutations of *ANGPTL4* lower plasm TG levels and reduce the risk of cardio vascular disease [201]. Hypoxic upregulation of ANGPTL4 and potentially increased TG levels in LE tissue may stimulate adipose tissue development.

Our studies on the effects of hypoxia on LECs also suggest a systemic function of the lymphatics on appetite regulation that has so far been completely ignored. In hypoxic LECs we observed significant down-regulation of the melanocortin-2 receptor accessory protein-2 (MRAP2) and up-regulation of melanin-concentrating hormone receptor-1 (MCHR1) (Fig. 11C; [143]). MRAP2 is a dominant-negative regulator of melanocortin-2 receptors [202], but enhances the generation of the second messenger cAMP when binding to melanocortin-4 receptor (MC4R). In hypothalamus, MC4R signals the feeling of saturation. Mice with global or brain-specific deletion of *Mrap2* exhibit severe early-onset obesity and mild hyperphagia [203–205]. In the human, *MRAP2* activity-reducing variants correlate with high body mass index [206]. MCHR1 is a G protein-coupled receptor up-regulated by hypoxia in LECs. *Mch1r*-deficient mice have reduced fat mass, which appears to be a consequence of hyperactivity and altered metabolism [207]. Of note, women with breast cancer-related arm lymphedema (LE) gain significantly more weight and are less active as compared to those without LE [208]. The potential regulation of appetite, locomotor activity and metabolism by LECs may open up a new field of LEC biology and research.

## Lymphatics and extracellular matrix (ECM) remodeling

In contrast to lymphatic collectors, initial lymphatics do not produce a continuous basal membrane. They are, however, connected to their microenvironment by anchoring filaments which are mainly made up of fibrillin-1 [25, 26, 32]. The glycoprotein EMILIN-1, which acts at the interface between elastin and fibrillin, supports the function of anchoring filaments. Its loss-of-function causes significant drop in lymphatic drainage [209]. In vitro, LECs produce not only elastin, fibrillin-1 and EMILIN-1, but also several typical components of a basal membrane, such as type-IV collagen, laminin, perlecan and fibronectin [160, 210]. During LE progression into stages-2 and -3, massive fibrosclerosis develops, and LE has therefore been regarded as a disease of the interstitial space. LE-induced fibrosclerosis can take place in any affected organ. In skin it is accompanied by a significant thickening of the corium, and, at the ultrastructural level, abnormal bundling and splicing of collagen fibers has been found [158]. Loss of elastin is another characteristic feature of LE [211]. Cells typically associated with fibrosclerosis are fibroblasts and myofibroblasts. So far, little or no consideration has been given to a possible function of LECs, although a basal membrane-like ECM aggregation develops around initial lymphatics in LE [158]. Experimental lymphostasis in dogs has revealed that hypoxia (anaerobic metabolism) is characteristic of the disease. The physiological pO_2_ of the dermis is in the range of 3% to 5% (24–35.2 mmHg) [212]. In vitro, chronic exposure of LECs to 1% pO_2_ (hypoxia) induces down-regulation of elastin (*ELN*), and upregulation of numerous genes involved in collagen production, bundling, and stabilization (Table 1; [143, 210]). In LE, it can be expected that fibrosclerosis in the immediate vicinity of the lymphatics is controlled by LECs.

**Table 1:** Genes upregulated by hypoxia in LECs

Gene name	Function
Long non-coding RNA H19	Fibrosis
Transforming growth factor-β 2, -3	Fibrosis
Prolyl 4-hydroxylase subunit alpha 1	Proline-hydroxylation
Procollagen-lysin 2-oxoglutarat 5-dioxygenase 2	Lysine-hydroxylation
Glucose transporter 3 (GLUT3/SLC2A3)	Vitamin C transport
Tensin-1	Myofibroblast differentiation
Lysyl-oxidase	Cross-linking of collagen filaments; copper-dependent
Ceruloplasmin	Copper transport
Fibromodulin	Cohesion of collagen fiber bundles
Tenascin-XB	Cohesion of collagen fiber bundles
Inter-alpha-trypsin inhibitor heavy chain family member 5	Inhibition of ECM degradation

## Conclusions

The functions of the lymphatic vasculature are much broader than generally thought, and even this review certainly does not list all of them. The phylogenetic and ontogenetic development alone still raises many questions about the lymphatics, their function and relationship to the blood vascular system. The physical connections between the blood and lymphatic vessels are not fully known. Even in the human, there certainly are more than just the connections to the ‘venous angle’. Studies on the molecular heterogeneity of lymphatic endothelial cells and smooth muscle cells in the wall of the collectors may serve as targets for the treatment of lymphedema by activation of collectors, and will reveal many more surprising clues to additional functions. Hypoxia-induced changes in gene expression in LECs may provide an indication of therapeutic options, e.g., for lymphedema.

## Data Availability

All data are included in the manuscript.
